# Shedding light on ultrasound in action: Optical and optoacoustic monitoring of ultrasound brain interventions

**DOI:** 10.1016/j.addr.2023.115177

**Published:** 2024-01-05

**Authors:** Maria Eleni Karakatsani, Héctor Estrada, Zhenyue Chen, Shy Shoham, Xosé Luís Deán-Ben, Daniel Razansky

**Affiliations:** a Institute for Biomedical Engineering and Institute of Pharmacology and Toxicology, Faculty of Medicine, University of Zurich, Switzerland; b Institute for Biomedical Engineering, Department of Information Technology and Electrical Engineering, ETH Zurich, Switzerland; c Department of Ophthalmology and Tech4Health and Neuroscience Institutes, NYU Langone Health, NY, USA

**Keywords:** Optical imaging, Optoacoustic imaging, Therapeutic ultrasound

## Abstract

Monitoring brain responses to ultrasonic interventions is becoming an important pillar of a growing number of applications employing acoustic waves to actuate and cure the brain. Optical interrogation of living tissues provides a unique means for retrieving functional and molecular information related to brain activity and disease-specific biomarkers. The hybrid optoacoustic imaging methods have further enabled deep-tissue imaging with optical contrast at high spatial and temporal resolution. The marriage between light and sound thus brings together the highly complementary advantages of both modalities toward high precision interrogation, stimulation, and therapy of the brain with strong impact in the fields of ultrasound neuromodulation, gene and drug delivery, or noninvasive treatments of neurological and neurodegenerative disorders. In this review, we elaborate on current advances in optical and optoacoustic monitoring of ultrasound interventions. We describe the main principles and mechanisms underlying each method before diving into the corresponding biomedical applications. We identify areas of improvement as well as promising approaches with clinical translation potential.

## GRAPHICAL ABSTRACT

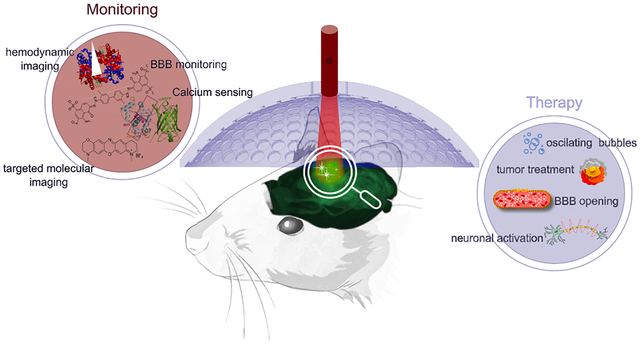

## 1. Introduction

The use of acoustic energy as a means to affect the brain dates back to the early days of medical ultrasound, when intracranial ablation was first introduced [1]. Ever since, therapeutic brain ultrasound advanced with technological developments in transducer design, precise calibration of acoustic power distribution, and research on the induced biological effects [2]. Modern high-intensity focused ultrasound (HIFU) systems enable precise transcranial delivery of a sufficient amount of energy to selectively heat the target tissue up to tens of degrees Celsius, which, combined with cavitation and other mechanical effects, leads to localized ablation within seconds without causing damage to surrounding areas [3]. Recently, new discoveries on alternative effects induced in the brain by focused ultrasound (FUS) at lower intensity levels have opened new possibilities to stimulate cerebral areas and circuits of importance in presently incurable neurological conditions [4]. FUS modes of actuation include transient blood–brain barrier (BBB) opening, neural activation, or temperature modulation. The basic mechanisms involved in these processes remain largely ununderstood. Thereby, new insights gained from an exponentially growing number of preclinical studies are poised to facilitate an eventual clinical translation of the basic biological findings. In parallel, significant efforts have been directed toward mitigating physical limitations imposed by the skull’s acoustic aberration and attenuation.

To this end, *in vivo* imaging has played an essential role in biological discovery [5], further enabling the study of fundamental mechanisms underlying ultrasound interaction with tissues [6] Several methods have been developed in the context of Magnetic Resonance-guided Focused Ultrasound (MRgFUS) therapy to characterize the effects of high intensity focused ultrasound on brain tissue [7] and monitor the onset and progression of thermal lesions [8]. Similar efforts have been made to study the subtle effects of low intensity ultrasound on various physiological mechanisms and targets, including BBB opening [9], brain activity [10], or gene delivery [11]. Optical and optoacoustic (OA) imaging techniques offer the unique capability to visualize dynamic processes *in vivo* with high molecular sensitivity and specificity, thus are particularly suitable to monitor neural and cerebral changes across different spatial and temporal scales. Optical contrast enables specific multiparametric characterization of hemodynamic changes, calcium and voltage activity, mapping transport of small molecules across the BBB, or screening cell transfection, to name a few examples [12,13]. The versatility of photon-tissue interactions is manifested by the large number of bioimaging methods capitalizing on optical contrast [14]. Furthermore, the hybrid OA modalities take advantage of a synergistic combination with ultrasound to enable high-resolution imaging with optical contrast in deep tissues under diffuse regime of light [15–17]. Overall, the multi-scale optical and OA interrogation of the brain can shed light into numerous physiological processes, ranging from activation of individual neurons and brain-wide connectivity all the way to thermal responses, BBB integrity, and modulation of glymphatic clearance.

Recent reviews summarized progress in the fields of ultrasound therapies [2–4,6,9] as well as optical [5,11,14] and OA imaging [15–17]. Herein we comprehensively review the use of light-based methods for monitoring the effects of FUS in the brain. We first consider the different mechanisms involved in FUS brain actuation as well as the capabilities of different imaging technologies. Subsequently, we report on preclinical studies combining the application of FUS with optical-contrast-based monitoring. The review concludes with a perspective on the clinical translation potential of these approaches and the remaining technical and application-related challenges.

## 2. Targeting deep into the brain with ultrasound

Ultrasound-mediated interventions have unique advantages over alternative therapeutic methods due to the physical characteristics of sound waves. Early applications of therapeutic ultrasound included treatments of uterine fibrosis, breast, prostate, and liver conditions [18]. FUS brain applications have emerged over the past decade owing to the progress in understanding the underlying interaction mechanisms as well as other technological and medical advances to overcome the shortcomings of transcranial ultrasound propagation. FUS is considered a non-invasive and non-ionizing technique that can penetrate deep into the brain and can be packed into a portable system [18]. Indeed, the clinical importance of brain FUS in neuroscience is growingly being recognized due to its unique capability to precisely focus ultrasound energy into targeted regions while minimizing exposure of surrounding tissues.

By taking advantage of mechanical and/or thermal effects induced by FUS in the brain, recent preclinical and clinical efforts have focused on the development of new interventions allowing new ways of tackling various clinically-relevant conditions, including, among others, movement disorders, chronic pain, epilepsy, and essential tremor [20,21]. The versatility of transducer designs (e.g. spherically-focused single elements versus multi-element phased arrays) as well as operating parameters (frequency, pressure, incidence angle, pulse cycle, duration, etc) enables superficial and deeper brain targeting with varying focal dimensions and intensity levels [22,23]. A range of brain regions, including cortical areas, the frontal lobe, the striatum, the hippocampus, and the midbrain have successfully been targeted. Therapeutic FUS at low frequencies could also be applied in the human brain, further underscoring the versatility of this technology and its ability to overcome anatomical variabilities across different species [24,25].

**Fig. 1.**
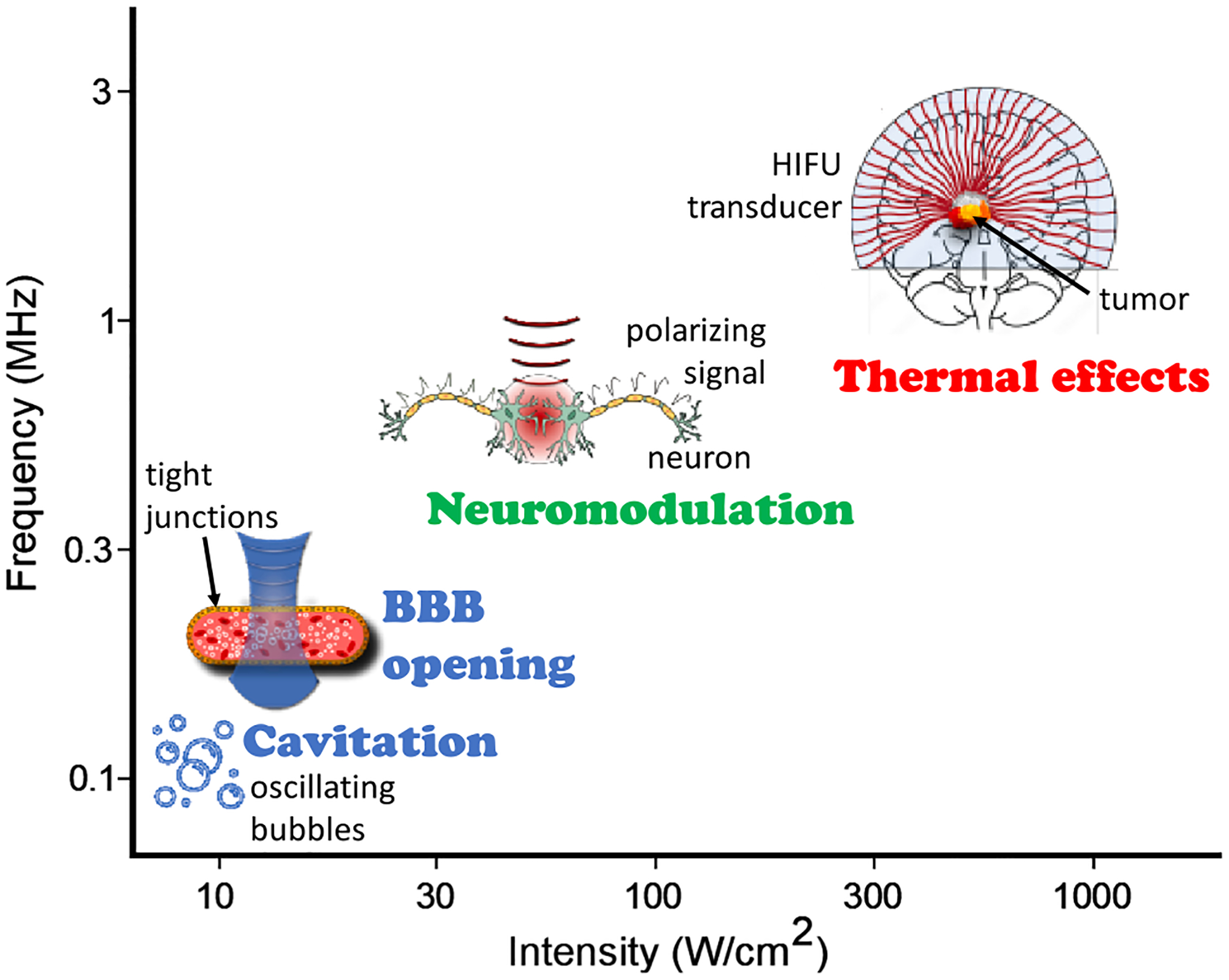
Intensity-frequency spectrum of the ultrasound mechanisms, namely cavitation, blood brain barrier (BBB) opening, neuromodulation and thermal effects.

Early FUS therapy took advantage of thermal effects emerging when ultrasound penetrates the tissue with part of its energy absorbed and converted into heat (Fig. 1) [18]. Ultrasound-induced thermal ablation is a non-invasive technique that induces cell death in a targeted area through temperature elevation, causing minimal collateral damage to surrounding tissues [18]. Thermal ablation has been used to treat brain disorders, such as essential tremor, neuropathic pain, and Parkinson’s disease [26,27]. Other thermal effects include increased tissue perfusion, dilated capillary size, and the enhancement of targeted drug delivery via thermo-sensitive carriers [28].

Another major effect is cavitation (Fig. 1), i.e., the formation of bubbles in a liquid resulting from negative (rarefactional) pressure induced by the propagation of acoustic waves. Primary mechanisms include stable cavitation, defined as controlled expansion and contraction of bubbles, and inertial cavitation, namely violent collapse of microbubbles further resulting in microstreaming, high pressure shock wave emissions, and free radical formation [29,30]. The bubble size and corresponding cavitation mechanism depend on the ultrasound frequency, the acoustic pressure amplitude, and the applied power. Cavitation events are more likely to occur at lower frequencies, and a large amount of energy is required to induce enhanced cavitational effects [29,30]. Cavitation is crucial in extracorporeal shock wave treatments and intracorporal lithotripsy, as well as for detecting dissected or fragmented tissue during surgery [30]. Cavitation bioeffects also include incremental cell permeability. For example, sonoporation refers to cell membrane permeabilization and drug uptake following stable FUS-induced cavitation of endogenous microbubbles [31]. Disruption of the tight junctions of the BBB, formed by endothelial cells surrounding brain vessels that hinder transport of molecules from the bloodstream into the brain parenchyma, could also be achieved with stable cavitation. BBB opening assisted with exogenous microbubbles has been extensively studied in the past decades [29,32] as they require only a fraction of ultrasound intensity to produce the desired effects. Such microbubbles stably oscillate under controlled ultrasound parameters and exert mechanical forces on the endothelial cells, thus causing structural and functional disruption of the tight junctions. They can also be fused together with therapeutic compounds or co-injected with pharmacological agents to enable controlled drug delivery [4]. The BBB has been successfully permeabilized in various animal species - from rodents to non-human primates - at precise locations within various brain structures. Clinically, several trials have recruited patients subjected to BBB opening, primarily in Alzheimer’s disease (AD) research [24].

Neuromodulation is a third effect induced by FUS that has recently gained importance as a non-invasive approach for neural stimulation (Fig. 1). Generally, neuromodulation is defined as the reversible excitation or inhibition of neurons or neuronal circuits [33]. This can alternatively be induced with electrical, chemical, cryogenic, magnetic, and light-based approaches [22,34–36]. Stimulating the brain using electrical methods renders robust results but unable to target deep brain structures without affecting superficial layers, unless implanted devices are being used. Non-invasive magnetic brain stimulation further suffers from poor spatial specificity, as it cannot be focused into specific brain regions. Using electromagnetic waves in the visible range of the light spectrum provides fast and precise stimulation of specific neurons labeled with light-gated ion channels. However, optogenetic neuromodulation cannot be performed non-invasively in humans as it involves genetic labelling of neurons and the use of visible optical wavelengths that are strongly attenuated within the human skin layers. Overall, the above neuromodulation approaches lack the precision and/or non-invasive deep-targeting capacity of ultrasound. FUS neuromodulation has been confirmed by various assays including behavioral analysis, calcium- and neuro-imaging *in vivo* as well as immunohistochemistry and electrophysiology [24]. Nonetheless, the underlying causes of FUS neuromodulation remain unclear, with proposed mechanisms ranging from activation of mechanically-sensitive voltage-gated channels, mechanical modulation of the membrane conductance, or intramembrane cavitation [6,37–40].

A critical aspect for successful therapeutic ultrasound is the real-time imaging feedback on the outcome of interventions. The MRgFUS therapy has propelled the use of therapeutic ultrasound for brain applications by providing both soft-tissue contrast and thermometry readings [24]. This has been essential in both treatment planning as well as real-time detection of the tumor-to-tissue interface, bone boundaries, changes in oxygen levels and perfusion rate, temperature elevation, tissue changes, and BBB opening with the administration of a contrast agent [24]. Ultrasound guided therapy, based on backscatter temperature imaging, has been implemented for HIFU ablation monitoring [41]. Estimation of the exposure time needed for a successful selective coagulation of tissue has also been demonstrated with high-resolution ultrasound thermography [41], where tissue deformations are captured by ultrasound speckle tracking. Conventional pulse-echo ultrasonography can detect increased echogenicity associated to lesion formation primarily due to bubble clouds, or tissue water boiling. “Listening” to microbubble oscillations differentiates stable cavitation characterized by harmonic and sub-harmonic frequencies from inertial cavitation associated to a broadband signal signature. Quantification of the cavitation dose has been used in real time monitoring of FUS-induced BBB opening in small animals and non-human primates [42].

The aforementioned monitoring approaches lack, however, the molecular specificity required to unravel basic mechanisms involved in FUS-based brain actuation, which is essential for streamlining clinical translation. The highly sensitive and specific interrogation of biological tissues with light gave rise to a myriad of applications with immense biological and clinical potential, as detailed below.

## 3. Functional and molecular specificity of light

**Fig. 2.**
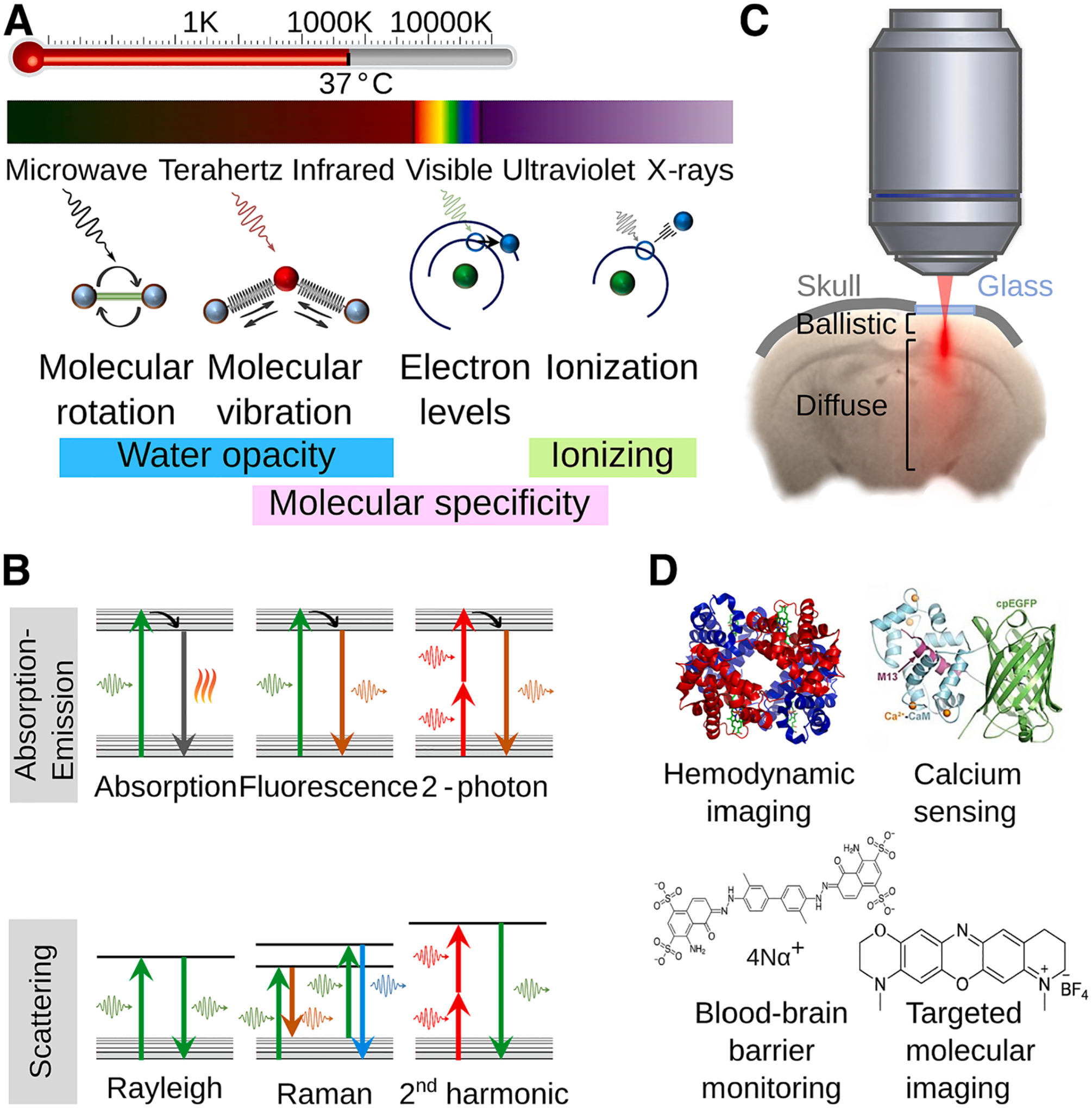
Optical imaging of the brain. A Mechanisms of interaction of photons with matter across the electromagnetic spectrum along with the black body emission peak for different temperatures. Spectral regions corresponding to strong absorption (opacity) of water, ionizing effects, and spectrally distinct photon-molecule interactions (molecular specificity) are also shown. B Jablonski energy diagrams corresponding to electron transitions involved during light absorption, emission, and scattering processes. The color of the arrows signifies the different light wavelengths involved in the transitions. C Propagation of light within the brain as a result of absorption and scattering events. The ballistic (single or a few photon scattering events) and diffuse (many photon scattering events) regimes are shown. A cranial optical window made of glass commonly used in microscopy techniques is shown in blue. D Examples of intrinsic and extrinsically administered molecules providing specific information on brain physiology and function.

In general, optical imaging technologies exploit specific photon-molecule interactions within biological specimens across different spectral ranges (Fig. 2A) [43,44]. Optical imaging modalities can be classified based on the type of contrast, resolution, acquisition time, depth range, or multiplexing capacity. A more general classification of optical methods used in biomedical research comprises two categories, namely *ex vivo* and *in vivo* modalities. *Ex vivo* methods are commonly used for imaging excised (fresh or fixed) tissue samples with superb resolution and sensitivity, while *in vivo* methods target non– or minimally-invasive imaging of living organisms. Histological analysis of tissue samples, mainly based on hematoxylin and eosin (H&E) staining, remains a workhorse in biological sciences [45,46]. Staining facilitates visualization of cellular morphology and tissue structure by capitalizing on color multiplexing, which refers to the simultaneous detection or visualization of multiple cell types, sub-cellular components, molecules, or other pathological or activity-based biomarkers [47].

Modern optical microscopy techniques can significantly outperform the limited multiplexing capability of traditional staining methods, thus massively enhance the amount of molecular information even without using chemical probes that may significantly alter the cell morphology and function. In particular, label-free molecular microscopy methods capitalize on interactions of photons with specific molecules to map their biodistribution with high specificity (Fig. 2B). A fundamental photon-molecule interaction is absorption, occurring in many biomolecules as a consequence of electronic transitions from ground to excited states. While absorption-based optical tissue interrogation in the ultraviolet and visible (UV–Vis) spectra [48] may provide valuable information on the distribution of nucleic acids, proteins, and other sub-cellular structures and molecules [49,50], single- and multi-photon excitation in the near-infrared (NIR) spectral range (800–1400 nm) is preferred to maximize the penetration depth of light into living tissues [43,51]. When moving further into the mid-infrared (MIR) region (2.5–20 um – 4000–500 cm^−1^), light attenuation in water severely limits the reachable depth but photon absorption is governed by very specific vibrational and rotational excitation of molecules (Fig. 2A). Black-body radiation emitted in this wavelength range also enables surface temperature mapping (Fig. 2A). MIR microscopy methods such as Fourier transform IR (FTIR) or discrete frequency MIR (DFIR) enable precise label-free visualization of nucleic acids, lipids, or proteins without the need of tedious sample preparation methods [52]. Labelling of small molecules with stable-isotope-based tags barely affecting molecular activity such as carbon-13 or deuterium can further enhance the MIR imaging capabilities e.g. for visualizing cell metabolism [52]. Another fundamental photon-molecule interaction is scattering. For instance, the basic mechanism of Rayleigh scattering underlies morphological imaging with epi-illumination bright-field microscopy, dark-field microscopy, or optical coherence tomography (OCT) modalities [53]. Molecular specificity is achieved with Raman scattering, resulting in Stokes and anti-Stokes photons with different wavelengths and excitation of vibrational molecular modes [54]. Raman microscopy approaches such as coherent anti-Stokes Raman scattering (CARS) [55], stimulated Raman scattering (SRS) [56], or coherent Stokes Raman scattering (CSRS) [57] can detect specific types of chemical bonds with high resolution and also exploit isotope-based and triple-bond-based tags to enable visualization of multiple small molecules with minimal perturbation [56].

Absorption and scattering also lie behind the optical opacity of biological tissues (Fig. 2C). Optical microscopy at cellular resolution is generally limited to a depth of ~ 100 μm. Even most advanced microscopes based on multi-photon excitation and adaptive optics can barely exceed the ~ 1 mm depth limit where light becomes fully diffusive [58]. Label-free MIR and Raman-based molecular imaging at shallow depths is commonly employed for elucidating the underlying mechanisms of cellular and molecular function [59,60]. Nevertheless, fluorescence imaging remains the mainstay of methods employed in biological discovery. It naturally occurs in certain molecules intrinsically present in tissues and cells, e.g. adenine dinucleotides, which has been exploited for label-free visualization of cellular metabolism with fluorescence-lifetime imaging microscopy (FLIM) [61]. However, fluorescence labeling is most commonly used to visualize cellular dynamics with specific fluorescent reporter proteins, small molecule dyes or nanoparticles [19,62,63]. Fluorescence microscopy can achieve high-resolution multiplexed imaging by capitalizing on the distinct absorption and emission spectra of fluorophores. Over the last decades, a myriad of advanced fluorescence microscopy embodiments have been developed, such as confocal [64], multi-photon [65], light sheet [66], light field [67], or the Nobel-prize-winning super-resolution [68,69] methods. Beyond the severe penetration depth limits, vast majority of high-resolution optical microscopy approaches are inapplicable in a human setting and further involve highly invasive procedures when it comes to imaging the rodent brain, such as scalp removal, craniotomy, and/or skull thinning.

On the other hand, the rich optical contrast has also motivated the development of meso- and macro-scopic molecular imaging modalities for deep tissue imaging with diffuse light and low spatial resolution, such as fluorescence molecular tomography (FMT) [70]. Apart from fluorescence, bioluminescence (light emitted by living organisms through chemical reactions) and the specific absorption spectra of oxygenated and deoxygenated hemoglobin are often used to retrieve functional and molecular information from the mammalian brain and other organs [71,72] (Fig. 2D). Indeed, deep-tissue optical imaging beyond the penetration limits of ballistic photons remains an active research area despite the limited resolution that can be achieved with diffuse light. This major drawback has recently been overcome with OA imaging methods merging light and ultrasound, as detailed in the next section.

## 4. Hybrid optoacoustic imaging

High performance monitoring of ultrasound interventions is fundamentally challenged by the need for i) high spatial and temporal resolution of the imaging modality, ii) simultaneous combination of FUS with imaging, and iii) high molecular sensitivity [73]. State-of-the-art imaging modalities fall short in achieving all three objectives. For instance, whilst offering high molecular specificity, optical imaging is unable to achieve high resolution for deep tissue observations. Conversely, ultrasound imaging renders high spatio-temporal resolution to the detriment of poor molecular contrast [74,75]. OA imaging capitalizes on a synergistic combination of optical excitation with ultrasound detection to render rich optical contrast from deep tissues with high spatio-temporal resolution [16].

The OA effect relies on the conversion of light to ultrasound energy through thermoelastic expansion and a subsequent local pressure rise [15,76,77]. Short-pulsed (<100 ns) lasers are typically used to excite endogenous tissue chromophores as well as extrinsically administered molecules or particles. Molecules excited with photons undergo radiative and non-radiative relaxation to return from higher to lower energy states. Tiny ultrasound waves in the megahertz-frequency range are then generated via non-radiative relaxation mechanisms, which can be detected with sensitive ultrasound transducers placed around the imaged object [77–79].

**Fig. 3.**
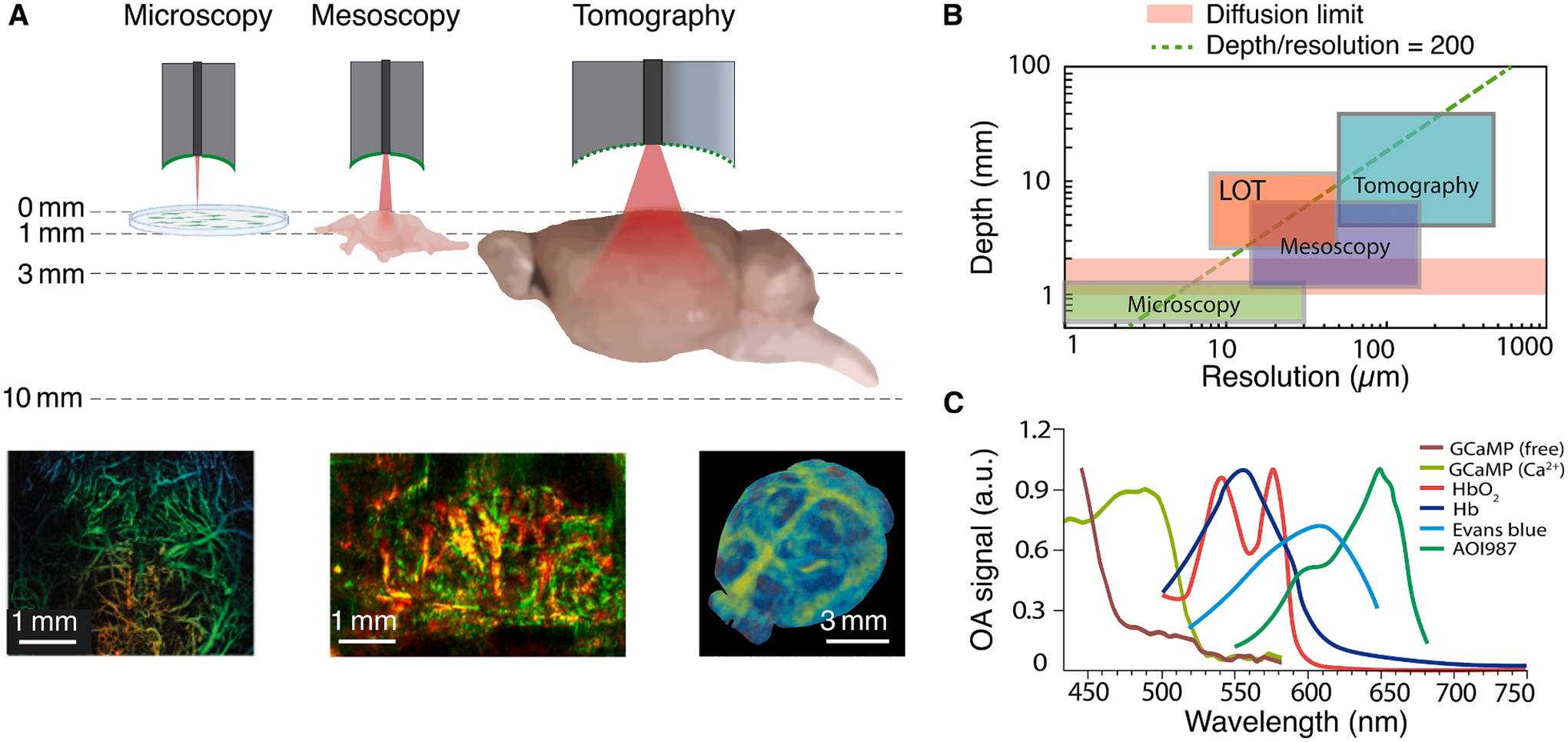
Optoacoustic (OA) neuroimaging. A Depth ranges covered by typical microscopic, mesoscopic, and tomographic (macroscopic) OA embodiments. Examples of *in vivo* mouse brain images obtained with these embodiments are shown. From left to right – large-scale OA microscopy of calvaria and brain vasculature (image reprinted with permission from [81]), raster-scan OA mesoscopy (RSOM) of brain tumors (image reprinted with permission from [82]), and whole-brain OA tomography of a mouse model of Alzheimer’s disease (image reprinted with permission from [83]). В Relationship between spatial resolution and depth covered by OA systems. LOT – localization optoacoustic tomography. C OA spectra of examples of molecules providing specific information on brain physiology and function.

OA imaging thus portrays the optical absorption characteristics of specific biological molecules [80]. OA imaging systems can be generally classified into microscopic [81], mesoscopic [82], and tomographic (macroscopic) [83] embodiments, depending on their spatial resolution, imaging depth, and field of view (Fig. 3A) [84]. This enables a high level of scalability when it comes to imaging the brain at various spatial and temporal scales, from individual cells and capillaries to the whole brain scale and from rapid temporal scale of neuronal signaling to slow longitudinal imaging of disease progression. The high (1–10 μm) lateral resolution provided by scanning optical-resolution OA microscopy is attributed to the tight focus of the laser beam at shallow depths. On the contrary, at depths where the optical beam is diffuse, high-bandwidth ultrasound sensors are used in scanning mesoscopy applications to capture deeper-located structures at the expense of inferior spatial resolution in the 20–50 μm range [85,86]. The unique multi-scale OA imaging capability is further complemented with OA tomographic imaging systems operating at depths of several millimeters to centimeters (whole-brain scale in mice). OA tomography is generally based on ultrasound arrays of hundreds of elements enabling simultaneous acquisition of a large number of signals with a single laser pulse, thus enhancing the imaging speed, effective depth, and field of view [87,88]. The microscopic imaging range can also be covered with custom-made, high-bandwidth arrays [89]. A mathematical inversion process accounting for wave propagation through the medium and sensor geometry is needed for tomographic inversions to accurately map location of the molecules of interest with high spatial precision (typically in the 100–200 μm range) [90–93]. Overall, OA imaging provides a uniquely broad coverage from shallow to deep tissues with the same type of contrast and a resolution typically scaling with 1/200 of the imaging depth [94] (Fig. 3B). In acoustic resolution embodiments, the resolution is limited by the ultrasound diffraction limit, which can be overcome with super-resolution imaging methods. For example, localization optoacoustic tomography (LOT) recently enabled deep-tissue microvascular imaging beyond the acoustic diffraction barrier by tracking circulation of highly-absorbing microparticles [95,96] (Fig. 3B). The advantages of absorption-based OA contrast are particularly manifested when considering multi-spectral (multi-wavelength) excitation. The use of tunable lasers or separate laser sources operating at different wavelengths facilitates multiplexed spectroscopic differentiation of multiple molecules featuring distinct absorption spectra [16,92] (Fig. 3C). The high-temporal resolution of OA tomography can also be used to capture dynamic changes in the optical absorption properties of specific molecules, e.g. due to photo-switching mechanisms [97,98] or variations in the physiological environment such as pH [99,100] or neural calcium dynamics [101]. Taken together, OA provides a unique five-dimensional (real-time spectroscopic three-dimensional) imaging capabilities, which are growingly exploited in preclinical and clinical studies [102].

To date, *in vivo* OA imaging of the brain has been achieved in different animal models, including rodents, larger animals, and non-human primates [103]. Initially, OA imaging was limited to cortical areas due to the limited penetration depth of light [104]. Internal illumination through the oral cavity enabled cross sectional imaging of deeper brain structures [103]. Recently, LOT has been shown to enable the visualization of deeper and finer brain vessels in the murine brain [96]. In addition to brain structure, vascular networks, hemodynamics, as well as functional and metabolic processes have also been imaged with OA systems. Stimulus-evoked brain activity could be detected through metabolic or cerebral responses as well as changes in optical absorption of genetically-encoded calcium indicators [105]. Functional brain connectivity can also be recorded by OA imaging of oxygenation dynamics [103]. Recently, multi-spectral OA imaging has been employed for the detection and monitoring of neurodegenerative diseases. For instance, the bio-distribution of targeted amyloid-binding AOI987 probe exhibiting distinct optical absorption spectrum in the NIR region could be visualized longitudinally, thus providing information on the AD’s plaque burden with ~ 110 μm spatial resolution across the entire mouse brain [106]. Imaging in the second NIR window, which offers the advantage of deeper penetration and increased SNR, holds great promise for Aβ detection. DMP2 was shown to preferentially bind to amyloid monomers in cortical and hippocampal sites, correlating well with immunohistochemical analysis [107]. On the other hand, tau is a second pathological hallmark of AD responsible for tauopathies and frontotemporal dementia. The pyridinyl-butadienylbenzothiazole derivative PBB5 probe has been shown to bind to tau and visualized with multispectral OA imaging in transgenic mouse brains [108].

The powerful OA imaging capabilities achieved in preclinical research pave the way toward clinical translation, primarily focusing on diagnostic and treatment monitoring applications. Diagnosis of breast cancer, dermatological disorders, microvascular perfusion, vascular dysfunction, or carotid stenosis are among many emerging clinical applications of OA imaging. The musculoskeletal system, adipose tissues and gastrointestinal structures could also be imaged with OA systems [109]. Altogether, the safe and non-ionization nature of OA coupled with the useable 2–5 cm imaging depth range, high spatial and temporal resolution, functional and molecular sensitivity outline a great range of potential clinical applications [109]. Human brain imaging has also been demonstrated with OA tomography in hemicraniectomy patients [110], albeit only limited penetration was achieved with transcranial imaging owing to the strong acoustic aberrations by the human skull [111].

## 5. Optical and optoacoustic monitoring of ultrasound interventions

### 5.1. Direct neuromodulatory effects

**Fig. 4.**
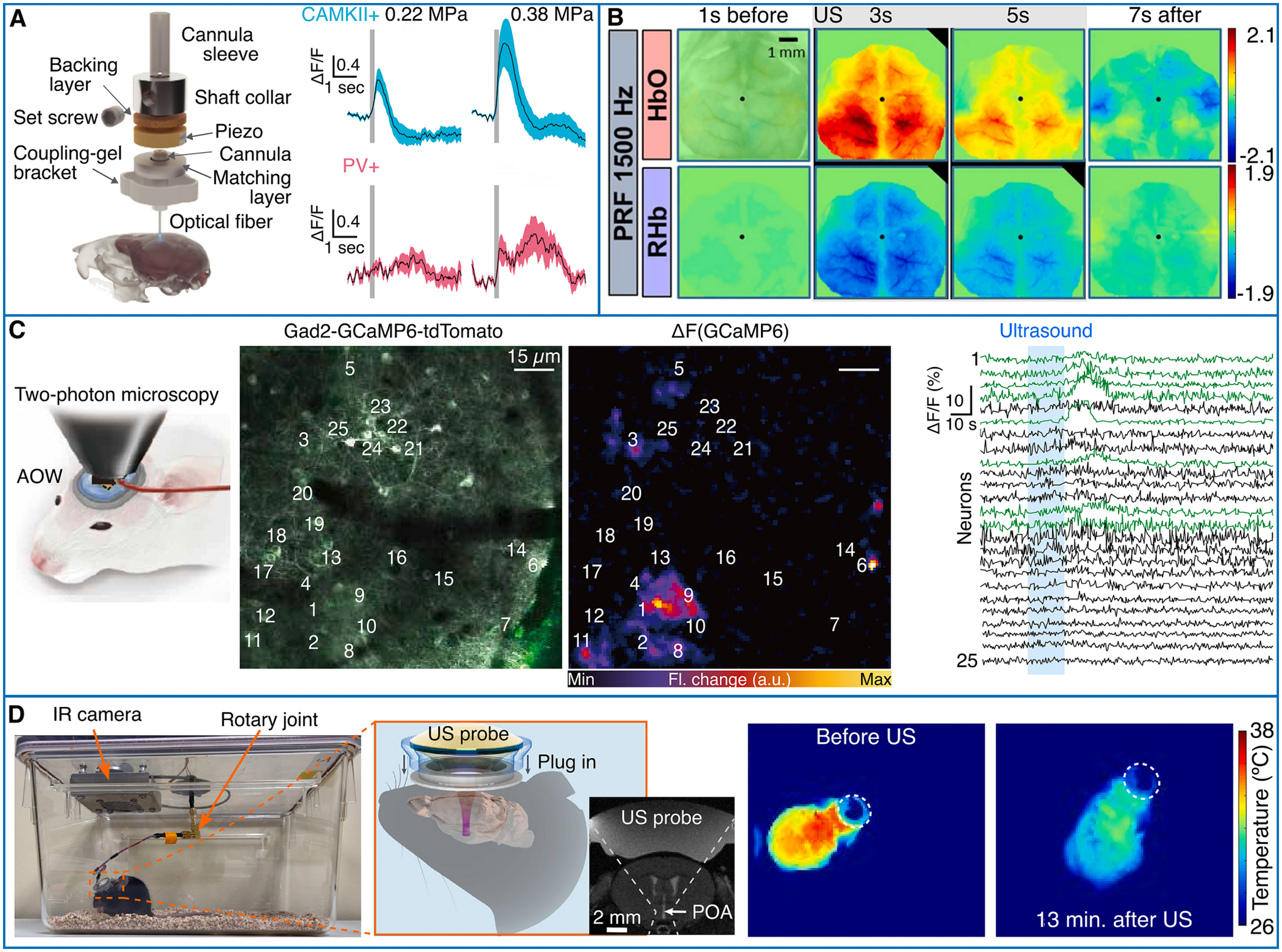
Optical monitoring of ultrasound neuromodulatory effects. A Integrated FUS and fiber photometry system allows the recording of fluorescence Calcium changes of Ca2+/calmodulin-dependent protein kinase II positive neurons (CaMKII + ) and parvalbumin positive interneurons (PV + ) of the hippocampus with increasing ultrasound pressure (200 ms pulse duration, shaded area: S.E.M.). Adapted with permission from [112]. B Spatial map of the hemodynamic concentration changes for oxygenated (HbO) and deoxygenated (HbR) hemoglobin elicited with ultrasound at 425 kHz, 200 ms pulse duration and 1.5 kHz pulse repetition frequency (PRF). Black dots indicate the position of the bregma while frames with black borders show statistically significant differences compared against sham stimulation. Reprinted with permission from x[124]. C Optically transparent ultrasound transducer integrated with two-photon microscopy. The acousto-optic window (AOW) is based on a coverslip and comprises a polyvinylidene fluoride-trifluoroethylene, piezo-polymer and indium-tin-oxide electrodes. The AOW is used as a cranial window and delivers an ultrasound stimulus at 10 MHz revealing the neural response at high resolution in the mouse cortex. Reprinted with permission from [118]. (center left) Two-photon fluorescence image at the somatosensory cortex of a Gad2-GCaMP6-tdTomato mouse and (center right) change in fluorescence (ΔF) due to ultrasound stimulation. Time traces (right) of the fluorescence change at the 25 neurons in the image. Time traces of neurons responding to ultrasound are plotted as green curves. D A wearable ultrasound device (left) targeted at the hypothalamus preoptic area (center left) triggers an hypothermic state that can be monitored using a thermal infrared camera (right) in behaving rodents. Reprinted with permission from [122].

As ultrasound neuromodulation involves multiple action mechanisms on neurons and the neurovascular unit (see section 2), real-time *in vivo* imaging plays a crucial role in elucidating the ultrasound effects on the brain. The optical opacity of conventional ultrasound emitters made of piezoelectric ceramics complicates efficient combination of ultrasound delivery with simultaneous optical imaging. Fiber photometry allows monitoring the responses to ultrasound stimuli in deep brain regions [112,113]. Despite being invasive, it allows monitoring calcium responses from different neuronal populations, highlighting their distinct responses to ultrasonic stimuli (Fig. 4A). However, this approach does not render an actual image with the calcium readout merely reflecting an integrated response from a volume located in the fiber’s proximity. Widefield calcium imaging offers a more precise way to assess neural activity by means of calcium sensitive proteins [114–117]. Other integrated systems have been reported for high resolution two-photon imaging [118] of ultrasound-triggered neural dynamics (Fig. 4C), allowing to record single neuronal responses through a cranial window [119].

Besides the basic science behind understanding how ultrasound affects neural activity, therapeutic applications in rodent models of epilepsy deployed integrated ultrasound delivery and sensing methods such as fiber photometry [112] and optical intrinsic imaging [120]. In addition, targeting deep brain structures in the brain with ultrasound has been shown to trigger metabolic and thermal changes in small rodents [121,122]. Inducing torpor-like state offers therapeutic prospects for mitigating brain damage after brain injuries, heart attack, or stroke, as well as for retarding cellular aging. The ultrasound delivery and imaging paradigm is then shifted to a wearable ultrasound transducer targeting the hypothalamic preoptic area and thermal imaging of the behaving animal using infrared cameras (Fig. 4D). Changes in body temperature and behavior can thus be assessed for long time periods in the range of 24 h.

#### 5.1.1. Hemodynamic responses

Neural activity and hemodynamic responses are linked through neurovascular coupling mechanisms, thus the latter are also induced by exposing the brain to ultrasound [123]. Optical intrinsic imaging [124–126] has mostly been used to monitor superficial ultrasound-induced hemodynamic changes (Fig. 4B) capitalizing on the different absorption properties of oxygenated and deoxygenated hemoglobin (Table 1). Less invasive than fiber photometry, it can be performed transcranially after the scalp is removed and only requires simple and accessible hardware. Hemodynamic changes induced by low-intensity transcranial ultrasound stimulation could be imaged with laser speckle contrast imaging (LSCI) [125,127]. It further revealed that low-intensity pulsed ultrasound (LIPUS) diminishes BBB disruption and edema formation and further induces a blood flow increase, thus indicating potential therapeutic benefits [128]. *Ex vivo* confocal microscopy images of DiI-stained microvascular networks corroborated a significant neuro-protection in mice exposed to LIPUS compared to untreated animals [129]. Terminal deoxynucleotidyl transferase dUTP nick end labelling (TUNEL) staining of the hippocampus and cortex further revealed reduced neuronal cell apoptosis [130].

Near-infrared spectroscopy based on the intrinsic optical contrast of hemoglobin has also been used to quantify hemodynamic responses to HIFU exposure [131]. It is also important to notice that radiation forces are also involved in endothelial nitric oxide synthesis, which induces vasodilation and hence hemodynamic changes [9]. *In vivo* imaging of ultrasound shock-wave induced nitric oxide generation was done with confocal microscopy by employing the nitric-oxide-sensitive diaminofluorescein-2 diacetate fluorescent probe (DAF-2 DA) [132].

#### 5.1.2. Glymphatic system

The glymphatic system consists of a distinctive network of perivascular channels created by astroglia cells to enhance the effective removal of soluble residues from the central nervous system and help distribute non-waste substances [133]. Being a relatively recent discovery, the glymphatic system function is not fully understood. Glymphatic drainage is impaired in neurological disorders such as AD, hemorrhage, stroke, or traumatic brain injury [134]. Restoration of glymphatic function can potentially play an important therapeutic role for treating these conditions. Recently, FUS has been shown to facilitate glymphatic clearance as confirmed by optical imaging of fluorescently-labelled fluid tracers (Table 1) [135,136]. Glymphatic drainage of beta-amyloid peptides was similarly improved in murine models of AD with microbubble-assisted FUS (Table 1) [137]. Fluorescence images of immunostained sections indicated a significant decreased deposition of beta-amyloid in treated mice. Mechanical manipulation of glymphatic transport via sonication of microbubbles was also verified with confocal microscopy images of fluorescently-labeled albumin in optically-cleared brain tissues [138]. Very low intensity FUS (<4 mW/cm^2^) was also shown to enhance glymphatic influx without injection of microbubbles, potentially leading to harmful effects [137]. *In vivo* transcranial fluorescence imaging of cerebrospinal fluid tracers demonstrated an influx increase at 15 min post stimulation (Table 1).

### 5.2. Mechanical effects

#### 5.2.1. Monitoring the blood brain barrier

Blood brain barrier (BBB) opening using microbubbles and FUS is an attractive alternative to HIFU-based brain tumor treatments commonly afflicted with undesired skull-induced heating and other transcranial focusing issues [139,140]. With the use of FDA-approved microbubbles, the BBB can be opened by stable cavitation of the microbubbles to deliver drugs into tumors or exert therapeutic effects in different neurodegenerative conditions [22,32]. Monitoring success of the BBB opening is crucial for the treatment outcome. In the lack of hemorrhages, intrinsic optical contrast is unable to assess the BBB integrity. OA imaging assisted with gold nanoparticles has been used to monitor their extravasation into brain tissue after BBB opening (Table 1) [141]. More sophisticated silica coated gold nanorods have been chosen for OA-guided BBB opening with FUS owing to their strong optical absorption contrast [142].

**Fig. 5.**
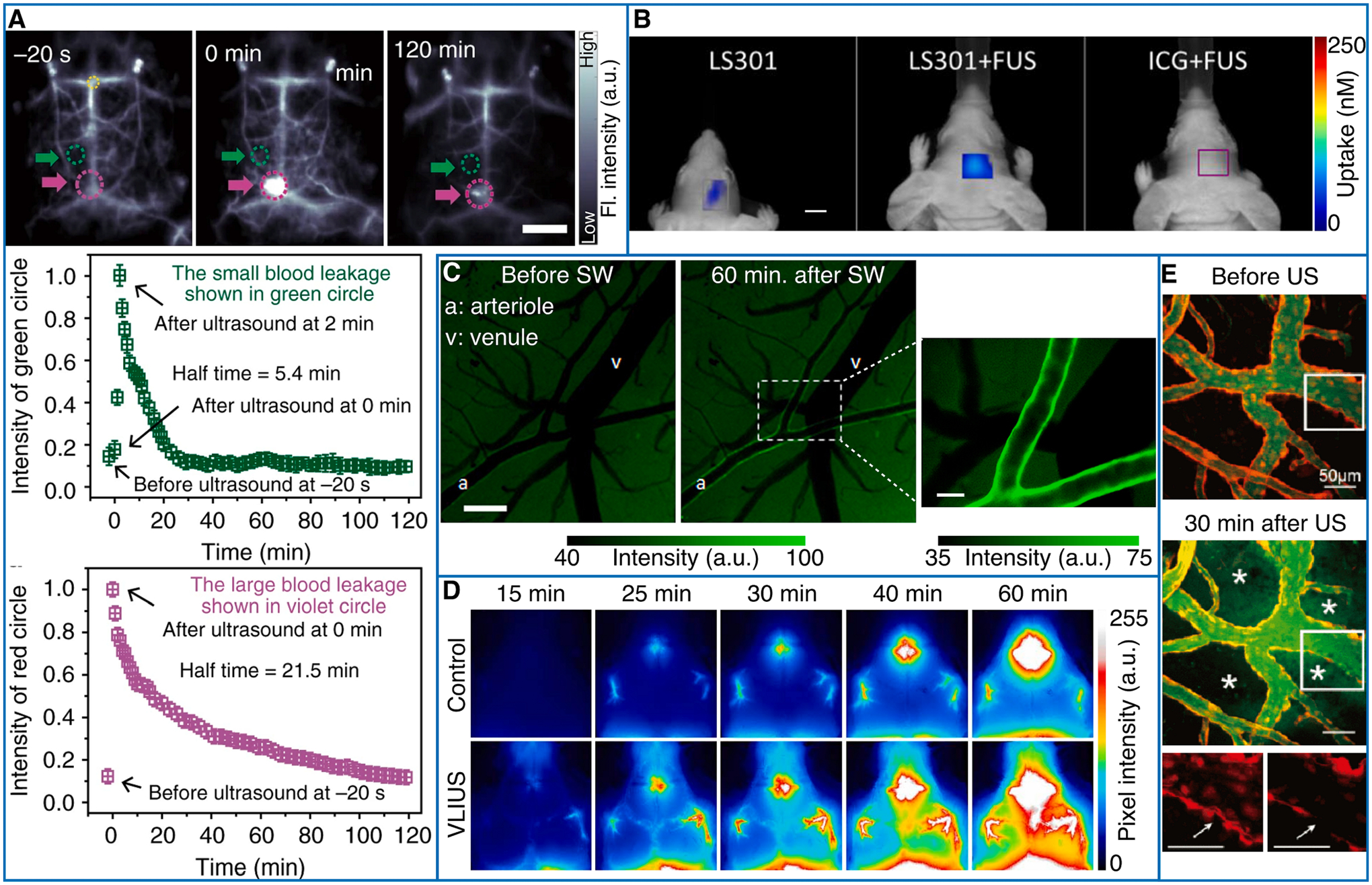
Optical monitoring of mechanical ultrasound effects. A Real-time non-invasive fluorescence imaging in the NIR-II spectral window to monitor the opening and recovery of the BBB. The images of fluorescence (Fl.) intensity in the mouse brain before and after FUS and microbubbles. Green and violet circles and arrows indicate the opening and recovering points of cerebral vessels which time evolution is shown in the curves below. Reprinted with permission from [143]. B FUS delivery of the tumor-targeting agent LS301 and indocyanine green (ICG) at 4 weeks post-tumor initiation monitored using FMT. Reprinted with permission from [145]. Colormap represents the uptake of the contrast agent. C DAF-2 T fluorescence in a rat cortex exposed to an light-induced shock wave (LISW). Reprinted with permission from [132]. D Time-lapse widefield fluorescence images of cerebrospinal fluid (CSF) influx over the first 60 min following albumin-Alexa Fluor 555 injection in control and very low intensity US-stimulated mouse. Reprinted with permission from [137]. E Two-photon maximum intensity projection images before and 30 min after FUS and microbubble treatment in a Tie2-cre::Ai14 transgenic mouse. Red channel: endothelial cells. Green channel: FITC-labelled dextran. Asterisk: Dextran diffusion in the interstitium; bottom panels: Magnified endothelial fluorescence images in the white box. Reprinted with permission from [146].

Another non-invasive approach takes advantage of the weaker light scattering at the NIR-II window to observe BBB disruption in relatively shallow brain areas using wide field fluorescence imaging (Table 1, Fig. 5A) [143–145]. Likewise, invasive microscopic approaches have been used to understand and assess the physiological effects of BBB opening [146] (Fig. 5E) as well as to test extravasation in tumor models (Table 1) [147].

OCT uses optical scattering and light interferometry to form an image. The methods is also sensitive to blood flow changes, which can be used to characterize the effects of FUS on microcirculation before and after sonication [148]. The detailed three-dimensional vasculature map produced by OCT with endogenous contrast makes it a compelling tool to observe microvasculature changes with high resolution, although simultaneous FUS delivery and OCT imaging has not yet been implemented.

#### 5.2.2. Drug delivery

Ultrasound-induced BBB opening has emerged as a targeted and non-invasive drug delivery technique, overcoming the drawbacks of conventional approaches. Monitoring of BBB opening, evaluation of drug release and confirmation of closure has been performed by several imaging techniques including optical and OA imaging [142,149]. Ultrasound-guided OA imaging has been validated by delivery of silica coated gold nanorods that can be localized due to their strong absorption capabilities [142]. Furthermore, dual-modality theranostic contrast agents have been developed by encapsulating therapeutic agents and chromophores into the microbubbles that disrupt the BBB under the application of ultrasound [149]. OA image-guided chemotherapy has been successfully performed in glioma bearing mice. Nanoparticles integrated with doxorubicin were delivered into the brain tumor following FUS-induced BBB opening under OA guidance (Table 1) [150].

HIFU methods have also been evaluated for controlled drug delivery to increase their therapeutic efficacy and reduce systemic toxicity (Table 1) [151]. Initially, HIFU was tuned to trigger the escape of a dye-loaded, phase-changing material from the gold nanocages and its release into the surrounding environment [152]. Alongside, doxorubicin was successfully released under application of HIFU and monitored by a raster-scanning OA mesoscopy (RSOM) system (Table 1) [151].

#### 5.2.3. Stem cell stimulation and differentiation

Several challenges remain to be addressed before the promising therapeutic prospects of stem cells can be efficiently exploited. At present, primary concerns include incomplete engraftment and viability at the transplantation site. LIPUS has extensively been used as an external biostimulation tool to accelerate tissue regeneration owing to its stimulatory effect on the function, proliferation, and differentiation of various cell types [153,154]. LIPUS increases angiogenesis and local blood perfusion; induces an immediate positive mechanical stimulation that enhances differentiation, proliferation, and maturation of many cell types, including chondroblasts, fibroblasts, and osteoblasts; and motivates the specific cell differentiation of mesenchymal stem cells (MSCs). Accordingly, several investigations have shown that direct and indirect mechanical stimulations play a primary role in the control of stem cell differentiation. Ultrasound exposure at appropriate intensity levels was reported to improve osteoblast maturation [155]. LIPUS was reported to have positive effects on chondrogenic differentiation of bone marrow-derived MSCs (BMSCs) [156] and maintenance of MSC stemness [157]. LIPUS was also shown to promote the migration of BMSCs and improve the fracture healing rate, while the intervention with focal adhesion kinase (FAK) and ERK1/2 inhibitors reduced the LIPUS-induced migration of BMSCs [158]. Systematic review on impact of ultrasound therapy on stem cell differentiation is available elsewhere [159].

### 5.3. Thermal effects

**Fig. 6.**
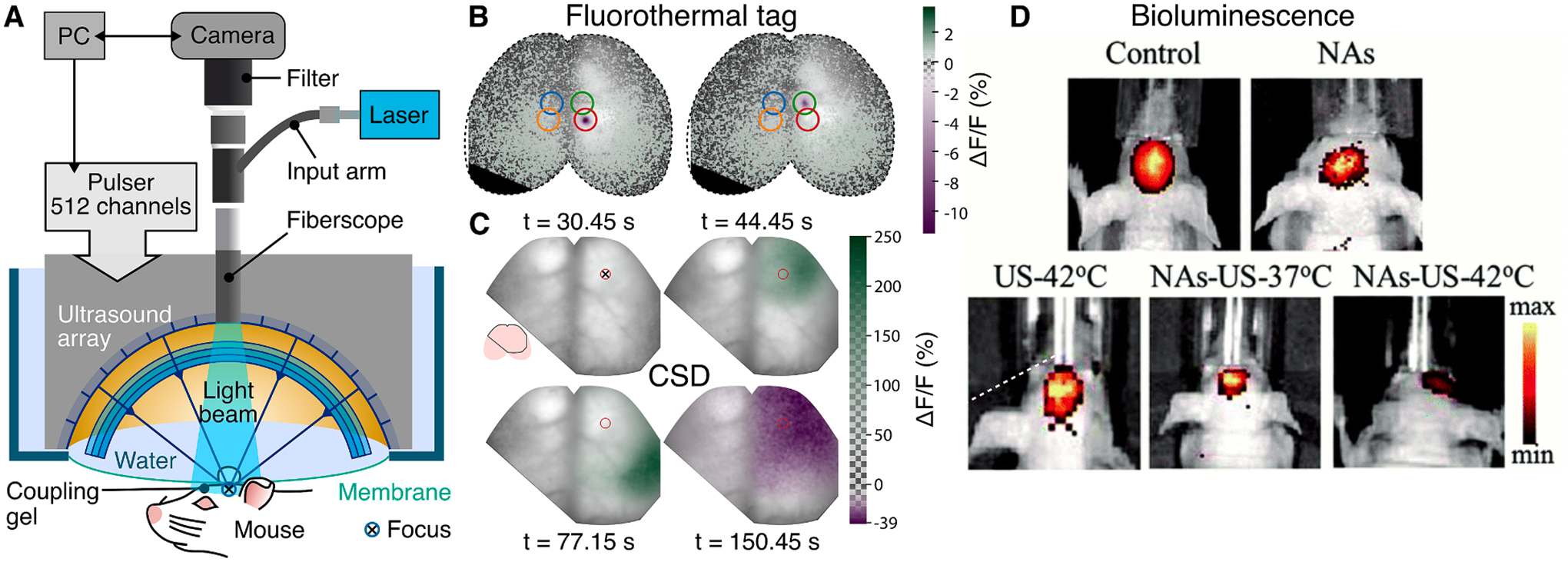
Optical monitoring of thermal ultrasound effects. A Wide-field fluorescence imaging integrated with high-precision transcranial ultrasound system. B Transient (150 ms duration at 3 MHz) thermal ultrasound effects can be monitored using fluorescence and be used as a tag to follow the position of the ultrasound focus on the GCamP6f mouse cortex. C Calcium imaging of thermally triggered cortical spreading depolarization (CSD). The red circle shows the point of ultrasound delivery. Reprinted with permission from [116]. D Bioluminescence images of mice with luciferase-labeled orthotopic U87 glioma after different treatments with thermally augmented sonodynamic therapy (SDT). Reprinted with permission from [165]. NAs: Nanoassemblies.

Temperature effects can quench fluorescence thus have been used to monitor temperature changes [160]. This effect has been exploited to monitor precise ultrasound delivery in the brain of GCaMP6f-expressing mice [116] (Table 1, Fig. 6A,B). Simultaneous widefield fluorescence and ultrasound delivery (Fig. 6A) can also report on physiological responses to ultrasound-induced thermal effects such as cortical spreading depolarization (Fig. 6C). Cortex-wide imaging of Ca2 + responses provides direct evidence of depolarization waves propagating throughout the mouse cortex.

OA imaging is similarly capable of monitoring temperature changes in tissue (Table 1) [161]. Its superior resolution and penetration depth over other optical modalities makes OA thermometry an appealing method for studying FUS-induced thermal effects. However, integration of high-power ultrasound transducers alongside sensitive transducers to detect the much weaker OA signals from tissue is not trivial. To this end, several approaches of OA-monitored HIFU have been proposed [162,163], although no OA thermometry in the brain has been reported.

Alternatively to HIFU, sonodynamic therapy (SDT) utilizes low intensity ultrasound combined with non-toxic sonosensitizers to induce reactive oxygen species that kill cancer cells, eventually suppressing tumor growth (Table 1) [164]. Bioluminescence of luciferase-labeled glioma tumor cells has been used to demonstrate the synergistic effect of moderate FUS-mediated temperature rise to 42 °C in the presence of sonosensitizer nanoassemblies [165]. (Fig. 6D).

## 6. Medical applications

### 6.1. Alzheimer’s disease

Presently, there is a growing interest in the application of FUS toward treatment of neurodegenerative diseases, both in preclinical models and human patients. Cerebrovascular dynamics of transgenic mouse model of AD has been studied with two-photon microscopy to monitor the ultrasound-induced BBB permeability *in vivo* (Table 2) [167,170]. Leakage kinetics of the TgCRND8 mice, which exhibit cerebral amyloid angiopathy, differs from that of healthy brains suggesting that amyloid-burdened vessels do not change their diameter upon FUS application. The same imaging approach was employed to follow the time-course of the FUS-induced BBB opening and amyloid plaques, concluding that a single FUS treatment reduces the plaque size while biweekly treatments is an effective therapeutic strategy for AD [171] (Table 2). Several groups have shown that FUS-induced BBB opening reduces the amyloid plaque load and eliminates tau from the brain of transgenic mice, further resulting in short term memory improvement of the treated animals. Confocal microscopy has been primarily used to provide information on the pathology biodistribution confirmed with analytical biomolecular assay [172–174].

Such beneficial outcomes paved the way for the first clinical trial showing successful BBB opening and closure in five AD patients [175] with several additional trials currently ongoing (NCT03739905, NCT03671889, NCT04118764) [173]. Among the latest advances in the field is the application of a transcranial pulsed stimulation (TPS) technique, which uses ultrashort ultrasound pulses for treating AD [176]. Several clinical trials over the past years showed that TPS may have a modulatory effect on the cortical thickness and atrophy of the stimulated regions [177]. Additional beneficial outcomes of brain TPS include anti-depressive effects and therefore this method could be expanded to all neuropsychiatric disorders [177,178]. Incorporation of *in vivo* optical or OA imaging into the treatment procedures may greatly contribute toward improving the therapeutic outcomes and understanding the basic mechanisms of TPS action on the brain.

### 6.2. Parkinson’s disease

The primary target in modifying the progression of Parkinson’s disease (PD) is the nigrostriatal pathway and the dopamine release. The progressive loss of neurons and the replacement dopamine has been demonstrated by the successful delivery of pharmacological agents (primarily including neurotrophic factors in the midbrain), which was facilitated by FUS-induced BBB opening [179]. PD studies have also shown significant improvements in motor abilities of mice that were treated with FUS-induced BBB opening assisted with curcumin-loaded nanobubbles [180]. An alternative to the direct protein delivery, gene therapy has gained popularity attributed to the constant release of a therapeutic protein and the specificity of the targeted FUS-induced BBB opening. The primary outcomes of these treatments include the assessment of behavioral changes and the detection of changes in dopamine release by *ex vivo* microscopy. In that sense, alpha-synuclein distribution in the brain, the integrity of the nigrostriatal pathway and dopamine levels in the affected brain regions, midbrain and striatum, have been examined with immunohistrochemistry [181]. Neurotrophic expression was demonstrated to occur up to 3 days after plasmid delivery through the BBB opening using *in vivo* epi-fluorescence imaging (Table 2) [182].

### 6.3. Bipolar disorders

Bipolar disorders are clinically complex, chronic and recurrent disorders. So far, few treatment options are effective across hypomanic, manic, depressive and mixed states and as continuation or maintenance treatment after initial symptom remission. By delivering the ultrasound energy to a specific brain region to increase or decrease the brain activity, low intensity focused ultrasound LIFU treatments could transform the quality of life and reduce the cost of care for patients with bipolar disorder. The feasibility and potential efficacy of LIFU in modulating amygdala function is currently being explored in a phase II clinical trial (NCT05228964). In terms of optical monitoring, OCT imaging revealed the thinning of the Retinal Nerve Fiber Layer (RNFL) in bipolar patients compared to healthy controls (Table 2) [183]. Lithium is extensively prescribed in patients suffering from mood disorders as it functions as a stabilizer. However, a lithium monitoring platform is essential given its narrow therapeutic window and low toxic dose. Preclinically, the feasibility of bipolar disorder monitoring was shown by employing lithium-sensitive nanosensors and spectroscopic OA imaging [184].

### 6.4. Stroke

Among cardiovascular diseases, stroke (ischemic and hemorrhagic) is of particular importance due to its neurological impact, as it may result in severe brain damage, long-term disability, and death. Ultrasound with intensities > 2 W/cm^2^ has been shown to facilitate disruption of blood clots based on mechanical effects [185]. This treatment, referred to as sonothrombolysis, can be enhanced with enzymatic activity or with cavitation induced by microbubbles [186]. Interactions of microbubbles with the thrombus have been studied with bright-field optical images taken by an ultra-high-speed camera at several million frames per second [187]. *In vitro* optical imaging also demonstrated hemolysis of clots post sonification [188]. The rich optical contrast has also been exploited to visualize changes in composition of blood clots during sonothrombolysis, i.e. the relative amount of red versus white blood cells. OA imaging has been used to monitor these changes in real time by capitalizing on its high spatio-temporal resolution [189]. Customized intravascular catheters for simultaneous delivery of high intensity ultrasound and light pulses for OA excitation have also been proposed [190]. Ultrasound stimulation at lower intensities further induces other therapeutic effects in stroke (Table 2). LIPUS has been shown to promote neurological recovery, mitigate inflammatory responses, and have a neuroprotective effect on brain injury [191] with optical imaging used to investigate these effects [192].

## 7. Clinical translation, outlook and perspectives

The synergistic benefits of combining light and ultrasound have been exploited in different biomedical fields [193]. Hybrid imaging modalities, such as acousto-optic tomography [194], ultrasound-modulated fluorescence imaging, ultrasound-assisted wavefront shaping [195], and, more prominently, OA imaging, have exploited the reduced scattering of ultrasound waves relative to photons within biological tissues in order to render high-resolution images of deep tissues with optical contrast. Ultrasound guidance of optical spectroscopic probes has also been proposed as an alternative means for rendering molecular-specific information with photons at ultrasonically-defined spots [196], whilst optical coherence elastography methods capitalize on ultrasound actuation to quantify tissue properties on the microscale [197,198]. The growing interest in ultrasound-based brain interventions calls for the development of new methods to enable the real-time monitoring during ultrasound actuation and comprehensive *in vivo* assessment of the outcome. Optical imaging modalities are poised to play an important role in fulfilling these needs as they are generally characterized by abundant and versatile contrast mechanisms and high imaging speed.

FUS-based brain therapies have recently attracted a growing attention of researchers, physicians, and the general public due to their promising new capabilities for clinical management of neurological diseases [24]. State-of-the-art therapeutic ultrasound technologies enable non-invasive and precise interventions, such as highly-localized thermoablation, drug delivery via transient BBB opening, and neuromodulatory brain stimulations. Much like for other species, the effects induced in humans depend on the ultrasound parameters such as frequency and intensity. Focusing of ultrasound energy into the human brain has been achieved with single-frequency excitation, bursts with controllable duty cycle, or short pulses generated with shock-wave systems [199]. Thermal effects, typically induced with relatively long exposures, are employed for tumor treatments and disruption of pathological brain circuits, e.g. for treating PD or essential tremor [200]. Mechanical forces are more prominently generated with short pulses or via stable microbubble cavitation [24]. The safety of microbubble-assisted BBB opening has been proven in multiple preclinical studies, and was also supported with initial clinical trials e.g. in AD or amyotrophic lateral sclerosis [173,175,201,202]. Ultrasound neuromodulation can also provide therapeutic effects in spite of the fact that underlying mechanisms remain unclear, as demonstrated in clinical studies in AD, depression, or disorders of consciousness [199].

A major advantage of FUS with respect to alternative tools for neurosurgery or brain stimulation therapies is that localized actuation can be performed in a non-invasive manner. However, transcranial propagation of ultrasound, severely affected by attenuation and aberration, is a major obstacle for efficient delivery of ultrasound energy into specific cerebral areas. Acoustic distortions are reduced at low ultrasound frequencies, thus clinical systems are typically based on transducers operating in the hundreds of kilohertz frequency range [203,204]. Currently, simulations based on X-Ray CT scans are the gold standard for compensating for skull aberrations [205]. These simulations commonly adjust the focus close to the central areas of the cranial cavity, whereas stirring away from the cranial vault’s central areas impairs the focusing performance [206]. Besides the ionizing radiation risks associated with X-Ray CT, its image resolution is insufficient for fully predicting transcranial ultrasound focusing, especially in areas adjacent to the skull.

Real-time monitoring of the interventions can be done with MRI. The excellent anatomical imaging performance of MRI along with its capability to detect temperature and hemodynamic changes have fostered the development of MRgFUS systems enabling simultaneous targeting, monitoring, and controlling the amount of energy delivered to the target spot [24]. MRI scanning, while a viable alternative, comes with several contraindications, such as the presence of magnetic materials in the body or the small bore size. Moreover, its widespread use is often impeded by the high procurement and maintenance costs, especially in low- and middle-income countries. The skull remains a significant barrier for the application of transcranial human brain investigations with OA imaging, with the majority of the generated high-frequency signals being attenuated and back-reflected into the brain, even in mice [207,208]. An advanced virtual craniotomy algorithm, developed for high-resolution OA imaging in living mice [209], employs a coregistered ultrasound image to correct for skull distortions. Yet, this method is not applicable in humans as it relies on transcranial light focusing. Alternatively, microbubbles and microabsorbers used for super-resolution ultrasound and OA localization may serve for guiding interventions [96,210]. Focusing at neighbouring regions can also be done by exploiting isoplanatism [211], while ultrasound localization microscopy has further been achieved in humans [212]. Most recently, transcranial OA brain imaging has also been demonstrated in humans [111,213]. It is thus expected that additional progress in transcranial OA neuroimaging will be achieved with skull aberration correction approaches, ranging from simple speed of sound corrections [214] to machine learning techniques [215,216].

Optical imaging of the brain is further challenged by the strong scattering of light. Optical microscopy can provide a comprehensive assessment of excised nervous tissues exposed to ultrasound *ex vivo*. High-resolution monitoring of the rodent cortical areas with state-of-the-art intravital optical microscopes also enables important insights into the basic mechanisms of FUS brain therapy. However, depths beyond a few hundred micrometers within the mammalian cortex are practically unreachable with intravital microscopy, even when using transparent optical cranial windows [217]. Yet, high precision targeting of deeper regions constitutes a major advantage of the FUS methods. Deep-tissue imaging with diffuse light strongly restricts the achievable spatial resolution, unless the induced effects are monitored right at the focal spot where spatial selectivity is achieved with ultrasound actuation. OA imaging can effectively mitigate the spatial resolution degradation with depth thus gains momentum as a powerful neuroimaging tool delivering multiparametric functional and molecular characterization of FUS-induced effects across the entire rodent brain. Despite the challenges related to bidirectional ultrasound and light transmission through the skull [218], new methods combining FUS delivery and real-time optical or OA monitoring may facilitate the clinical translation of newly-developed ultrasound therapeutic approaches.

Optical and OA brain imaging chiefly relies on the endogenous contrast provided by hemoglobin in red blood cells. Diffuse optical mapping of brain activation exploits the spectrally-distinctive absorption spectra of oxygenated and deoxygenated forms of hemoglobin to detect oxygen consumption and blood flow changes. Advances in near infrared spectroscopy (NIRS) and diffuse optical imaging (DOI) provide functional information matching that of functional MRI (fMRI), the gold standard in human neuroscience [218,219]. Merging NIRS or DOI with FUS delivery presents an exciting avenue for exploration. For this, independently operating optical and ultrasound sensors need to be efficiently hybridized, which is challenged by the lack of ultrasound transparency of the former and optical transparency of the latter. Nevertheless, NIRS monitoring of FUS therapy using existing technology may become instrumental in monitoring broad cortical responses to stimulation of remote brain structures and replace electroencephalography in situations where electrical crosstalk with ultrasound devices constitutes a major problem [220]. Early studies have shown that OA signals from vascular structures in the human brain could be detected [221]. The feasibility of transcranial OA tomographic imaging has further been deployed with recent technological advances [111,213]. Transcranial OA imaging of the brain is affected by skull-induced aberrations in a similar manner as FUS, albeit to a lesser extent than in pulse-echo ultrasonography that involves bidirectional propagation of the acoustic waves. A combination of OA tomography and ultrasound delivery, as demonstrated in rodents [222], holds promise for monitoring shallow cortical brain regions in humans, which can become a valuable tool for navigation and functional neuroimaging provided that the physical barriers are mitigated with further developments. High-resolution angiographic OA imaging of the brain was achieved in craniectomized patients [110], which demonstrates the great potential of this approach if the signal acquisition approaches and reconstruction algorithms can effectively be adapted to correct for transcranial ultrasound aberrations [211]. On the other hand, an acoustically-matched polymeric material has been proposed for cranial ultrasound windows in patients undergoing reconstructive skull surgery [10], which may also be used to facilitate OA monitoring of FUS treatments.

Optical-contrast techniques have been consistently employed in the field of neurosurgery during tumor resection procedures with new approaches, e.g. based on optical harmonic generation microscopy, FLIM, or Raman spectroscopy, being recently introduced [223–225]. Fluorescence-guided brain surgery is also an active field of research with new targeted and unspecific contrast agents being constantly developed [223–225]. Intraoperative ultrasound has also been used for monitoring brain tumor surgeries [226]. The integration of intraoperative ultrasound therapy could usher in a new era inside the operating room provided its effectiveness in disease prevention and treatment is demonstrated. In this context, neurosurgeons may benefit from having additional tools to extend their reach beyond the brain’s surface with rich optical contrast to monitor effective FUS delivery with high precision and sensitivity.

In this review, we covered the integration of optical and OA monitoring with therapeutic brain ultrasound into a single platform to enable a wide range of emerging applications. This approach is rapidly gaining ground with a multitude of new technological developments emerging, as manifested by the growing number of research studies capitalizing on the powerful optical contrast to monitor ultrasound interventions. Future directions of this dual-modality approach include development of novel theranostic agents, endoscopic techniques to enable deep tissue imaging during FUS delivery, as well as overall system miniaturization and cost reduction. From the signal and image processing perspective, development of algorithms for real-time feedback monitoring, overcoming skull-induced acoustic aberrations, super-resolution imaging, noise cancellation, as well as introduction of machine-learning-based approaches are all expected to result in an enhanced performance. We thus expect that these recent advances in optical and OA monitoring of ultrasound brain interventions will accelerate the transformation of these primarily experimental techniques into routine clinical practice.

## Figures and Tables

**Table 1:** Methods, parameters, and findings of representatve *in vivo* studies grouped per ultrasound mechanism.

Mechanism	Intervention	Optical/OA imaging	Ultrasound	Perspective	Citations
Neuromodulation	Hemodynamic changes	Intrinsic signal optical imaging (ISOI), 465 and 560 nm, 33 Hz for each wavelength	Transcranial ultrasound operating at 0.425 MHz in pulsed mode having the same intensity	ISOI has simple and accessible hardware and achieves high spatio-temporal resolution, though at shallow penetration depth	[124]
		Laser speckle contrast imaging (LSCI), 635 nm, 20 mW, 20 ms exposure time	BBB opening and edema treatment with LIPUS, center frequency 0.5 MHz	LSCI is a simple, cost-effective and label-free technique that can achieve perfusion images over large field of view with tens of micron spatial resolution	[125]
		Intrinsic signal optical imaging (ISOI), CWL = 610 ± 2 nm	Transcranial ultrasound at 0.5 MHz, stimulation duration 300 ms and maximum ultrasound pressure 0.55 MPa, corresponding to 10.1 W/cm^2^	Time-frequency pattern modulation of cerebral blood oxygenation and neurovascular coupling	[126]
	Glymphatic system	Fluorescence imaging with fluorescent solute tracers ovalbumin (45 kDa and fluorescein (2000 kDa) isothiocyanate (FITC)-dextran	Improving glymphatic clearance with FUS operating at a 0.2 MHz fundamental frequency in pulsed manner for 30 min	Glymphatic clearance can be monitored *in vivo* and in real time with fluorescently labelled tracers, though only superficially	[135,136]
		Fluorescence imaging with albumin- Alexa Fluor^™^ 555 as the CSF tracer	LIPUS treatment was performed at center frequency 1.0 MHz, and various spatial peak temporal average intensities (Ispta) = 0.92, 3.68, and 5.85 mW/cm^2^	Glymphatic clearance can be monitored *in vivo* and in real time with fluorescently labelled tracers	[137]
Mechanical effects	Blood-brain barrier	OA with a 15-MHz probe (680, 970 nm) and 3D color Doppler (3DCD) to detect FUS-induced BBB opening	FUS-mediated (1.68 MHz) indocyanine green nanoparticle (ICG/NPs) delivery	ICG-labelled MBs combined with FUS could be used to open and synchronously visualize the BBB. Transformation of ICG-MBs into lipid-ICG NPs under FUS irradiation could enhance their ability to penetrate and accumulate in the brain tumor. OA signal depends on the ICG concentration. Effect size, dye concentration, and maximum absorbance on NP pattern distribution	[141]
		OA with a NIR-II molecule (LZ-1105) with absorption and emission beyond 1000 nm. Enhanced with long blood circulation time, continuous real-time monitoring of dynamic vascular processes, including cerebrovascular imaging, opening and recovery of the BBB	FUS-induced BBB opening at 0.5 MHz frequency, 0.6 MPa acoustic pressure, and 20 s sonication duration	OA is capable of reporting the activity of customized contrast agents over a wide range of near infrared (NIR) wavelengths, yet only for superficial vessels	[143]
		NIR-I/NIR-II fluorescence imaging (Ex, 745 nm; Em, 840 nm) and a NIR-II fluorescence imaging system (Ex, 808 nm, power density, 60 mW/cm^2^)	FUS-induced BBB opening at 1.0 MHz frequency, 0.28, 0.36, and 0.46 MPa acoustic pressures for 2 min	Real-time monitoring of the opening and recovery of BBB. Cerebral vascular structures clearly visible at a depth over 1.3 mm under the intact scalp and skull	[144]
		Two-Photon Intravital Imaging with FITC-dextran (500 kDa) at 920 nm excitation	FUS-induced BBB opening at 1.85 MHz frequency, 0.43 MPa acoustic pressures for 90 s	Dynamic process of FUS/MB-mediated Dextran extravasation across the BBB. Vasoconstriction and vasodilation occurred frequently upon ultrasound sonication. However, a cranial window is necessary	[146]
		Two-Photon microscopywith Texas-Red dextran at 920 nm excitation	Ultrasound-induced BBB opening at 1.85 MHz frequency, 0.43 MPa acoustic pressures for 90 s	Temporal profile of dextran extravasation and gradual accumulation in the tumor interstitium	[147]
		Label-free optical coherence tomography (OCT) and angiography (OCTA) at 1060 nm	FUS exposure at 0.4 MHz frequency for 120 s at power levels 1, 2, 2.5 and 5 W, equivalent to 0.153, 0.216, 0.242, and 0.343 MPa	Microstructure and microcirculation observation. Blood leakage detection Optimization of the applied FUS exposure power Vessel dilation in different exposure conditions. Vascular effects increased with the applied acoustic pressure. Excessive FUS exposure power degrades the OCTA signal	[166]
		Two-photon imaging with Dextran-conjugated Texas Red	FUS-induced BBB opening at 1.15 to 1.2 MHz for 120 s with 0.26 to 1.45 W power, corresponding to 0.071–0.25 Mpa	Real time monitoring of BBB opening by dye leakage only in the treated brains. Three leakage responses were identified, namely, fast, sustained and slow, depending on the applied acoustic pressure	[167]
	Drug delivery	OA imaging of ultrasmall Cu_2−*x*_Se nanoparticles attached to the surface of nanoparticles encapsulating doxorubicin (DOX) in the NIR (808 nm) window	FUS transducer with 1 MHz center frequency and 0.3 MPa acoustic pressure	The developed multifunctional theranostic nanosystems exhibit tumor-triggered programmed destruction. Controlled destruction of the shell and release of therapeutics. The nanoparticle biodegradation behaviour is concentration dependent	[150]
		IVIS Spectrum CT imaging of Cy7-labeled doxorubicin-capsules	HIFU transducer with center frequency 2.75 MHz delivering 1.94 W/cm^2^ energy	Destruction of the particles results in Cy7 leakage, detectable by optical imaging and doxorubicin for anticancer therapy. Carrier loading affects fluorescence. Temperature impacts particle aggregation, and drug release	[151]
		Multispectral OA imaging of AuNRs in the 700–950 nm window	FUS transducer with 2.0 MHz center frequency	US-guided contrast-enhanced OA for therapy monitoring	[168]
		Multispectral OA tomography based on ultrasmall Cu_2x_Se nanoparticles	FUS transducer with center frequency 0.5 MHz and acoustic pressure 0.6 MPa	Monitoring nanoparticle release and clearance and the induced immune response. Anticancer drug release monitoring	[169]
Thermal effects	Heating	Fluorescence imaging at 488 nm	HIFU-mediated temperature rise, 150-ms duration pulses at 3 MHz delivered sequentially every 11 s onto two different locations in the mouse cortex	Fluorescence signal is sensitive to temperature changes. Integrated wide-field fluorescence imaging and ultrasound delivery system allows the simultaneous observation and discernment between thermally and ultrasonically induced tissue responses in the same experiment	[116]
		OA imaging at 680 nm, 10 Hz frame rate, and 7.4 mJ/cm^2^ per pulse energy	HIFU-induced thermal effects at a center frequency of 1.5 MHz, and power of 50 W	OA thermometry can provide a linear proportionality between the OA amplitude and the measured temperature, and most of the data was within ± 10 % of the trend line tolerance. The temperature increase of surrounding tissue might affect the OA amplitude	[161]
		Bright field optical imaging with Evan’s Blue dye injection	Sonodynamic therapy (SDT). Tumors were treated with ultrasound at 1.0 MHz	Image-based analysis renders Evans blue perfusion. Presence of dye was semi-quantified by determining the distribution of dark pixels in the image	[164]

**Table 2:** Medical applications of ultrasound therapy monitored by optical/OA imaging tools.

Application	Optical/OA imaging	Ultrasound Therapy	Perspective	Citations
Alzheimer’s Disease	Two-photon microscopy with fluorescent dextran (70 kDa) Texas Red 810 nm excitation wavelength	FUS-induced BBB opening with 1.15–1.30 MHz center frequency range, 0.4–0.8 MPa acoustic pressure range and 120 s duration	The kinetics of the BBB leakage from intact vessels in the TgCRND8 brain appeared qualitatively different than in the healthy brains Acoustic pressure correlates with leakage The effect of plaque presence on the BBB kinetics of BBB remains unknown	[170]

	Two-photon microscopy with fluorescent dextran (70 kDa) Texas Red 900 nm excitation wavelength Methoxy-X04 750 nm excitation wavelength	FUS-induced BBB opening with 1.1 MHz center frequency, 0.4–0.8 MPa acoustic pressure range and 120 s duration	One FUS treatment reduces the size of existing β-amyloid plaques for two weeks Repeated biweekly FUS treatments is an effective method of reducing β-amyloid pathology in moderate-to-late stages of AD Dextran leakage into the extravascular space affected the necessary laser power needed	[171]
Parkinson’s Disease	Bioluminescence imaging (IVIS-200)	FUS transducer with 0.5 MHz center frequency, power range 0.8–5.4 W (equivalent to negative pressure 0.3–0.8 MPa) and 60 s duration	Significant increase of GDNF/GFP gene expression, neuroprotective effects and restoration of PD-model motor behavior	[182]
Stroke	OA (PRR = 10 Hz, 532 nm excitation wavelength, ~8.3 mJ/cm^2^ energy density)	Sonothrombolysis at 0.5 MHz spherically focused, single element transducer with 0.64 MPa pressure	OA can monitor blood clot changes in real time by capitalizing on its high spatio-temporal resolution. The OA SNR reduces after treatment due to the presence of the microbubbles	[183]

## Data Availability

No data was used for the research described in the article.

## Abbreviations:

FUS: High- focused ultrasound
HIFU: High intensity focused ultrasound
BBB: Blood-brain barrier
OA: Optoacoustic
MR: Magnetic resonance
MRI: Magnetic resonance imaging
MRgFUS: Magnetic-resonance-guided focused ultrasound
H&E: Hematoxylin and eosin
UV–Vis: Ultraviolet–visible
MIR: Mid-infrared
NIR: Near-infrared
DFIR: Discrete frequency
OCT: Optical coherence tomography
CARS: Coherentanti-Stokes Raman scattering
CSRS: Coherent Stokes Raman scattering
FLIM: Fluorescence-lifetime imaging microscopy
FMT: Fluorescence molecular tomography
LIPUS: Low-intensity pulsed ultrasound
BM MSC: Bone marrow-derived MSCs
FAK: Focal adhesion kinase
TPS: Transcranial pulsed stimulation
LIFU: Low intensity focused ultrasound
LSCI: Laser speckle contrast imaging
TUNEL: terminal deoxynucleotidyl transferase dUTP nick end labelling
DAF-2 DA: Diaminofluorescein-2 diacetate
AD: Alzheimer’s disease
PD: Parkinson’s disease
RNFL: Retinal nerve fiber layer
SDT: Sonodynamic therapy
DOI: Diffuse optical imaging
